# Nicotine Negatively Affects Its Users’ Health and Psychology in Saudi Arabia: A Cross-Sectional Study

**DOI:** 10.3390/healthcare14030286

**Published:** 2026-01-23

**Authors:** Jehad A. Aldali

**Affiliations:** Department of Pathology, College of Medicine, Imam Mohammad Ibn Saud Islamic University (IMSIU), Riyadh 13317, Saudi Arabia; jaaldali@imamu.edu.sa

**Keywords:** nicotine pouches, effects on health, psychological impact, Saudi Arabia

## Abstract

**Background**: Recently introduced nicotine pouches (NPs) are smokeless nicotine products. They are held between the lips and gums for 30 min to absorb nicotine into the bloodstream through the oral mucosa. Attractiveness may increase nicotine use, especially among young people and teens. The objective of this study is to investigate the health issues and psychological effects associated with nicotine pouch use among individuals in Saudi Arabia. **Methods**: A cross-sectional online survey using Google Forms. It was conducted between 13 February and 4 November 2025, in the Riyadh province of Saudi Arabia, restricted to users of nicotine pouches willing to answer a questionnaire on the occasion of buying them (at regional tobacco stores/supermarkets) or online via WhatsApp or the Telegram platform. Statistical analysis was conducted using SPSS Version 27, with a *p* < 0.05 indicating significance. **Results**: The current study included data of 489 participants, with a total of 395 participants using nicotine pouches. The most commonly reported symptoms were difficulty breathing and shortness of breath (both 40.5%), changes in taste or smell (36.7%), headache and stomach ulcers (33.4% each), and rapid or irregular heartbeat (28.4%). Most common psychological symptoms at any severity level (slightly to extremely) were appetite changes (78.7%), difficulty concentrating or focusing (75.4%), difficulty sleeping (74.9%), and increased anxiety or irritability (73.4%). Depression (72.2%), anger management (71.1%), and stress (70.4%) were also common. Regression analyses revealed that educational attainment was a significant predictor of both physical and psychological health outcomes. **Conclusions**: The findings show the most common physical symptoms were difficulty breathing and shortness of breath, followed by taste or smell changes, headaches, stomach ulcers, and rapid or irregular heartbeat. Appetite changes, concentration issues, sleep disturbances, and anxiety or irritability were common across all severity levels. Depression, anger issues, and stress were common.

## 1. Introduction

Smoking is a major contributor to various health issues, including life-threatening cardiovascular diseases, debilitating lung cancers, and chronic obstructive pulmonary disease [1]. Cigarette smoking is the primary method of nicotine consumption in many demographics and regions, despite its negative effects on nearly every organ in the body [2]. Nicotine is released into cigarette smoke during combustion. The natural appeal of smoking reinforces its addictive appeal. High nicotine levels in tobacco plants attract users due to its their potent psychoactive effects [3]. Nicotine quickly permeates the bloodstream during a smoking session, causing its effects to spread throughout the body [4]. Nicotine binds to neuronal nicotinic receptors, regulating mood and promoting relaxation [5], with a sudden increase in plasma nicotine levels followed by a decrease [6].

Smokeless nicotine products, such as oral nicotine pouches (ONPs), are gaining popularity [7]. Typically, nicotine pouches are small and white and measure about 25 mm in length, with nicotine concentrations generally between 3 and 10 mg. These pouches are designed for discreet placement between the gums and lips for 30 min, which allows nicotine to be absorbed through the oral mucosa and enter the bloodstream, releasing it gradually without combustion [8]. These pouches contain carefully selected components, which are diverse. Nicotine pouches may include various flavors, such as mint, citrus, and fruit blends, to meet diverse user preferences in addition to nicotine, the main active component [9]. Additionally, alkaline salts are often added to adjust the pH level of pouch contents. This adjustment enhances nicotine absorption and product effectiveness [9]. Nicotine consumption may increase due to increased attractiveness, especially among previously unfamiliar populations like young people and teenagers. Nonetheless, NP is marketed as a lower-risk alternative method of quitting smoking; however, its long-term health effects are not entirely understood in Saudi Arabia [10].

A 2020 study by Sushanth et al. examined 416 male construction workers in Chennai, India, and identified a significant correlation between nicotine dependence and symptoms of anxiety and depression [11]. This trend raises concerns regarding the potential impact of oral nicotine pouches on health problems and psychiatric alterations; consequently, oral nicotine pouches remain a relatively novel product in Saudi Arabia, and their effects on mental and digestive health are not yet well comprehended. In contrast to cigarettes, which have been extensively studied over the years, these pouches have garnered minimal scientific attention [12].

In 2023, Dowd et al. published some preliminary results [4]. Oral lesions were observed in nearly half of the 118 adult users. Additionally, 39% experienced gastrointestinal distress, 37% oral pain, 21% throat pain, and 9% nausea. These results stress the need for more studies examining the possible dangers to human health from these products, especially with regard to their effects on the digestive system and general well-being. Nicotine, the active component in nicotine pouches, is known to have both physical and psychological side effects [13]. On the other hand, conventional tobacco products and oral nicotine pouches are entirely different. Their unique delivery method and different ingredients might have varying effects on health due to the different biochemistry approaches for entering the body. It is still mostly unclear how they affect the gastrointestinal system, particularly in terms of gut motility, bloating, and long-term digestive problems. To the same extent, their impacts on mental health need more targeted research [14].

In 2023, Saudi Arabia introduced nicotine pouches, which sparked both interest and concern about their possible effects on users [12]. The gap is particularly evident in Saudi Arabia, where social norms and cultural practices influence both the use and perception of nicotine pouches. Our hypothesis posits that individuals utilizing nicotine pouches may encounter gum irritation, gastroesophageal reflux disorders, and effects on psychological well-being. To assess this, we are investigating the health problems and psychological effects of nicotine pouch use among individuals in Saudi Arabia.

## 2. Material and Methods

### 2.1. Study Design and Settings

This cross-sectional study was performed at the Department of Pathology, College of Medicine, Imam Muhammad bin Saud Islamic University, Riyadh, Saudi Arabia.

### 2.2. Study Participants

Residents of Saudi Arabia who were 18 years of age or older and had used nicotine pouches within the previous six months were recruited. To maintain the sample’s focus and mitigate bias, we did not exclude non-residents, first-time, or occasional users but individuals with a pre-existing psychological or health condition were excluded. Furthermore, non-nicotine pouch users and minors aged less than 18 years were excluded. Participants were restricted to completing the survey on only one occasion, a procedure to prevent duplicate submissions.

### 2.3. Ethical Approval

This research was reviewed and approved by the committee of the Institutional Review Board (IRB) at Imam Mohammad Ibn Saud Islamic University. The project identification number for this research was 757–2025, and it was completed on 12 February 2025.

### 2.4. Sample Size Calculation

The size of the study’s sample was determined with the help of the power calculator Raosoft (Raosoft Inc., Seattle, WA, USA). The province of Riyadh contains approximately 1214 people who use NP. For the purpose of achieving a confidence level of 95% with a margin of error of 5%, it was determined that a sample size of 385 participants was sufficient. However, a minimum sample size of 385 participants was determined using standard cross-sectional study parameters.

### 2.5. Study Questionnaire Development

A cross-sectional study was conducted in the Kingdom of Saudi Arabia between 13 February and 4 November 2025, through an online survey on Google Forms in Arabic and English.

The survey intentionally targeted individuals who have used nicotine pouches to report any side effects or psychological impact they have experienced as a result of their use. The survey was disseminated through social media platforms (Telegram and WhatsApp) and stores that sold nicotine pouches (supermarkets and tobacco stores). Prior to participating, participants were required to submit electronic consent. Demographic data, including gender, age, nationality, and marital status, was collected in the initial section of the survey.

The second section of the survey gathered information regarding the nicotine dosage and the types of previous nicotine products used by the participants. The third section of the survey was designed to gather responses from participants who experienced adverse effects as a result of using the pouches. Participant responses to the pouches’ physiological effects were collected in the fourth section of the survey. Participants’ perceptions of health effects were assessed using a composite Health Effects Score.

### 2.6. Bias

We took measures to minimize bias by utilizing a validated tool, removing individuals with known comorbidities that could have impacted the results, and anonymizing responses.

### 2.7. Statistical Analysis

SPSS version 27 was used for analysis. Data was presented using descriptive statistics, and bivariate and multivariate logistic regression analysis identified associations between nicotine pouch use and healthy or psychological symptoms. Associations between nicotine pouch use and categorical variables were assessed using Pearson’s chi-squared or Fisher’s exact test as appropriate. Multivariable logistic regression was performed to identify independent predictors of nicotine pouch use, reporting odds ratios (ORs) with 95% confidence intervals (CIs). Linear regression was used to explore associations between sociodemographic factors and both health and psychological effects, reporting beta coefficients and 95% CIs.

## 3. Results

### 3.1. Demographic Characteristics

A total of 491 responses were obtained. Nonetheless, two respondents chose not to participate. Therefore, the present study comprised data from 489 participants. Ninety-four participants who did not utilize NP were excluded. Table 1 presents the demographics of 395 nicotine pouch (NP) users derived from a study involving 489 participants. Male (96.45%) and Saudi (93.67%) users constitute the majority of the data. The population is predominantly young, with 48.6% aged 18 to 25 and 30.63% aged 26 to 35. Despite their relative youth, 75.94% of participants possessed Bachelor’s degrees, and more than 11% had completed postgraduate studies. More than half of the participants were daily smokers (52.91%), while 34.93% reported smoking occasionally. Only 12.15% of the participants indicated that they had never smoked.

### 3.2. Characteristics of Nicotine Pouch Use

A total of 395 participants had ever used nicotine pouches, representing 80.8% of the sample. Among the users, the majority reported daily use (61.3%), followed by 25.3% who used them less than daily but at least once a week. The most commonly reported symptoms were difficulty breathing and shortness of breath (both 40.5%), changes in taste or smell (36.7%), headache and stomach ulcers (33.4% each), and rapid or irregular heartbeat (28.4%). Most users had consumed nicotine pouches for less than 5 years (72.4%), and the most common dosage was 7–10 mg (61.8%). Half of the users (50.9%) used nicotine pouches anytime, while smaller proportions reported using them with friends (15.9%) or while working or studying (8.9%). This summary is presented in Table 2.

### 3.3. Factors and Predictors of Nicotine Pouch Use

In the univariable analysis, gender and age were significantly associated with nicotine pouch use (*p* < 0.05). In the multivariable logistic regression model, female participants had significantly lower odds of using nicotine pouches compared to males (OR = 0.17, 95% CI, 0.07 to 0.38, *p* < 0.001). Compared with those aged 18 to 25 years, participants aged 46 to 55 years (OR = 0.26, 95% CI, 0.09 to 0.74, *p* = 0.011) and those older than 55 years (OR = 0.15, 95% CI, 0.03 to 0.77, *p* = 0.019) were less likely to use nicotine pouches. Everyday smokers were 4.57 times more likely to use nicotine pouches than individuals who have never smoked, while occasional sometime smokers were 3.65 times more likely to use them. There is an everyday link between smoking history and nicotine pouch use. This summary is presented in Table 3.

### 3.4. Health Effects of Nicotine Pouch Use

**Figure 1:**
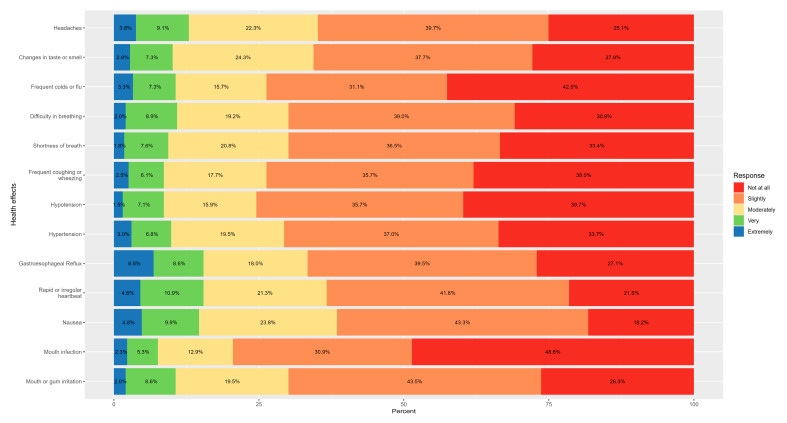
Health effects of using nicotine pouches.

Among nicotine pouch users, the most frequently reported health effects at any severity level (slightly to extremely) were nausea (81.8%), rapid or irregular heartbeat (78.5%), headaches (74.9%), mouth or gum irritation (73.7%), changes in taste or smell (72.2%), and gastroesophageal reflux (72.9%). Additional commonly reported symptoms included difficulty in breathing (69.1%), hypertension (66.3%), frequent coughing or wheezing (62.0%), hypotension (60.3%), and mouth infection (51.4%). See Figure 1.

### 3.5. Psychological Effects of Nicotine Pouch Use

**Figure 2:**
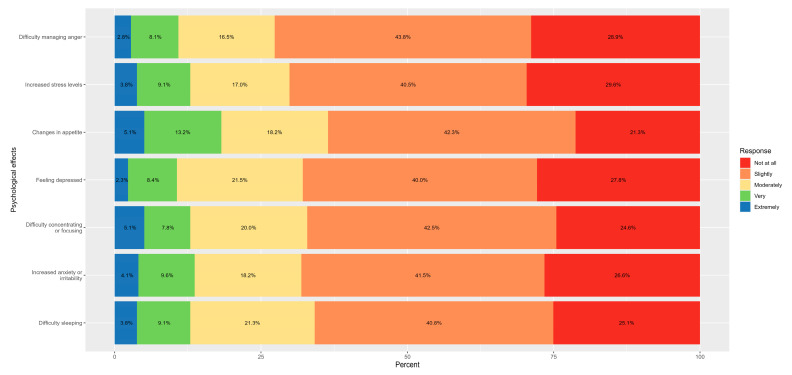
Psychological effects of nicotine pouch use.

The most commonly reported psychological symptoms at any severity level (slightly to extremely) were changes in appetite (78.7%), difficulty concentrating or focusing (75.4%), difficulty sleeping (74.9%), and increased anxiety or irritability (73.4%). Feeling depressed (72.2%), difficulty managing anger (71.1%), and increased stress levels (70.4%) were also prevalent. See Figure 2.

### 3.6. Health and Psychological Effects of Nicotine Pouch Use Among Users

In the multivariable linear regression model, education was significantly associated with health effect scores. Compared to participants with no formal education, those with a high school education (beta = −9.93, 95% CI, −16.0 to −3.82, *p* = 0.002) and those with a Bachelor’s degree (beta = −5.65, 95% CI, −11.3 to −0.03, *p* = 0.049) had significantly lower health effect scores. Smoking status is a very strong predictor (*p* < 0.001). See Table 4.

The psychological effects of education were significantly correlated with psychological effect scores. Compared to never-smokers, participants who smoked occasionally (beta = −2.90, 95% CI, −4.79 to −1.01, *p* = 0.003) and those who smoked daily (beta = −3.35, 95% CI, −5.14 to −1.55, *p* < 0.001) had significantly lower psychological effect scores. In addition, participants with a doctoral degree had significantly higher scores compared to those with no formal education (beta = 5.60, 95% CI, 0.71 to 10.5, *p* = 0.025). No significant associations were observed for gender, nationality, age, or marital status in the multivariable analysis. See Table 5.

### 3.7. Factors Associated with Mouth or Gum Irritation

Mouth or gum irritation was significantly associated with marital status (*p* = 0.045) and education level (*p* = 0.044). A higher proportion of married participants (79.1%) reported irritation compared to unmarried ones (70.0%). Regarding education, participants with doctoral degrees had the highest prevalence of irritation (91.7%), while those with high school education had the lowest (54.8%). No significant associations were found for gender, nationality, or age. See Table 6.

### 3.8. Factors Associated with Mouth Infection

Mouth infection was significantly associated with age (*p* = 0.005), marital status (*p* = 0.002), education level (*p* < 0.001), and smoking status (*p* = 0.018). The prevalence of infection increased with age, from 42.2% among those aged 18 to 25 to 83.3% among those above 55. A higher proportion of married participants reported mouth infections (60.8%) compared to unmarried ones (45.1%). Participants with doctoral degrees had the highest prevalence (100%), while those with high school education had the lowest (33.3%). See Table 7.

## 4. Discussion

Nicotine pouches are gradually gaining recognition as a socially acceptable alternative nicotine product, primarily owing to their perceived safety and social advantages. They are generally regarded as safer than conventional cigarettes, as they do not contain tar, produce smoke, or involve combustion [13,15]. Although their usage has been increasing in popularity, the potential health effects of nicotine pouches, including gastrointestinal and psychological disturbances, continue to be a concern, especially in countries such as Saudi Arabia, where research is limited. The current study aims to fill this knowledge gap by exploring the prevalence of health and psychological effects of nicotine pouch use among the population of Saudi Arabia.

A total of 489 volunteers completed the survey. The participants consisted predominantly of Saudi nationals (male and female). A substantial proportion of participants (395) had used nicotine pouches at least once. The participant nicotine pouch types: 97% DZRT, 2.5% VELO, and 1% ZYN. Among these users, most reported daily use, with a smaller group indicating weekly but less-than-daily use. The symptoms most frequently described included difficulty breathing and shortness of breath, changes in taste or smell, headaches, stomach ulcers, and rapid or irregular heartbeat. The findings agree with those of Al-Nafisi et al. [14]. Most users had been consuming nicotine pouches for fewer than five years, and the dosage most frequently selected fell within the seven to ten milligram range. Half reported using nicotine pouches at any time throughout the day, while smaller segments used them socially or during work or study. Approximately half of the respondents were in the youngest age group (18–25), with the next largest proportion in early adulthood (26–35). Most were unmarried and held a Bachelor’s degree. Nearly half reported smoking daily, while about one-third smoked occasionally. There is a very strong link between smoking history and nicotine pouch use.

In univariable analyses, gender and age displayed significant associations with nicotine pouch use. Multivariable logistic regression revealed that women had substantially lower odds of using nicotine pouches compared with men; the findings agree with those of Elsokkary et al. [12]. Older age groups, particularly those in midlife and older adulthood, also had markedly lower odds of use relative to the youngest group. This finding is consistent with a Saudi study by Aldhahir et al. [16]. Non-smokers who use nicotine pouches may report or experience more significant health changes/effects than those who are already accustomed to nicotine through smoking.

Among users, the most commonly reported physical health effects, across any level of severity, included nausea, rapid or irregular heartbeat, headaches, mouth or gum irritation, changes in taste or smell, and gastroesophageal reflux. Additional frequently noted symptoms were difficulty breathing, hypertension, coughing or wheezing, hypotension, and mouth infections [12,14]. Psychological symptoms were also highly prevalent, with changes in appetite, difficulty concentrating, sleep disturbances, increased anxiety or irritability, depressed mood, difficulty managing anger, and heightened stress levels all reported by substantial proportions of users.

In the multivariable linear regression analysis for physical health effects, education was a significant predictor. Participants with a high school education or a Bachelor’s degree had markedly lower health effect scores than those with no formal education. Regarding psychological effects, education was a significant factor, with participants with a doctoral degree exhibiting higher scores than those without formal education. No significant associations emerged for gender, nationality, age, or marital status in these models.

Specific symptoms demonstrated additional associations. Mouth or gum irritation was significantly related to marital status and education, with married participants and those holding doctoral degrees more likely to report this symptom. The findings agree with those of A Mishra et al. [4]. Mouth infection was associated with age, marital status, and education. Its prevalence increased across age groups, was higher among married individuals, and was greatest among those with doctoral degrees. Those with lower levels of education consistently reported the lowest prevalence of both irritation and infection. The findings agree with those of Al-Nafisi et al. [14]. No significant associations were identified for gender or nationality.

### Limitation

The research in Saudi Arabia aims to examine the health and psychological effects of nicotine pouches on users. It has multiple limitations that should be considered. Initially, the cross-sectional design prevents establishing causality between nicotine pouch use and the observed outcomes, thereby limiting the study to associations rather than definitive conclusions. Furthermore, the validity of the findings may be affected by the presence of biases, such as recall bias or social desirability bias, stemming from the dependence on self-reported data.

Another concern is residual confounding, which arises when not all potential confounders have been accounted for, potentially leading to biases in the observed associations. Furthermore, the findings may have limited applicability to other populations as the study is specific to the context of Saudi Arabia, and the impact of nicotine pouches in different regions could be affected by cultural, social, and regulatory variations. Furthermore, it is important to acknowledge that our study is susceptible to selection bias, as the survey was conducted online, potentially excluding individuals without internet access or those who do not use social media, thereby potentially limiting the generalizability of the sample. Self-selection bias may also be present, as individuals who took the time to respond might have held more pronounced opinions or experiences related to nicotine pouch use, potentially influencing the results.

## 5. Conclusions

A broad range of physical health effects was frequently reported, as well as psychological symptoms. Regression analyses indicated that education level was a significant predictor of both physical and psychological health outcomes, while gender, nationality, age, and marital status generally showed no significant associations, except in the case of specific symptoms such as mouth irritation and mouth infection, which were more prevalent among married individuals, older adults, and those with higher educational attainment.

## Figures and Tables

**Table 1:** Demographics of 395 nicotine pouch (NP) users.

Characteristic	Description n (%)
**Gender**	
Male	381 (96.45%)
Female	14 (3.54%)
**Nationality**	
Saudi	370 (93.67%)
Non-Saudi	25 (6.32%)
**Age**	
18 to 25	192 (48.60%)
26 to 35	121 (30.63%)
36 to 45	52 (13.16%)
46 to 55	24 (6.07%)
Above 55 years	6 (1.51%)
**Marital status**	
Married	157 (39.74%)
Unmarried	238 (60.25%)
**Education**	
Not educated	9 (2.27%)
High school student	41 (10.37%)
Bachelor’s Degree	300 (75.94%)
Master’s Degree	34 (8.60%)
Doctoral degree	11 (2.78%)
**Smoking status**	
Never smoked	48 (12.15%)
Sometime smoked	138 (34.93%)
Every day smoke	209 (52.91%)

**Table 2:** Characteristics of nicotine pouch use (n = 395).

Characteristic	Description
**Frequency of using nicotine pouches**	
Daily	242 (61.3%)
Less than daily, at least once a week	100 (25.3%)
Less than once a week, at least once a month	35 (8.9%)
Symptoms *	
Changes in taste or smell	145 (36.7%)
Difficulty concentrating	107 (27.1%)
Headache	132 (33.4%)
Stomach ulcers	132 (33.4%)
Rapid or irregular heartbeat	112 (28.4%)
Skin problems (e.g., premature aging/yellowing of fingers/teeth)	37 (9.4%)
Chronic sinus infections or bronchitis	82 (20.8%)
Frequent colds or flu	46 (11.6%)
Difficulty breathing	160 (40.5%)
Frequent coughing or wheezing	110 (27.8%)
Shortness of breath	160 (40.5%)
**Number of units**	
Less than 5 years	286 (72.4%)
5–10 years	95 (24.1%)
More than 15 years	14 (3.5%)
**Dosage**	
1–3 mg	35 (8.9%)
4–6 mg	70 (17.7%)
7–10 mg	244 (61.8%)
More than 10 mg	46 (11.6%)
**Timing of use**	
At home	34 (8.6%)
With friends	63 (15.9%)
While working/studying	35 (8.9%)
After exercising	0 (0.0%)
When bored	32 (8.1%)
When tense/stressed	30 (7.6%)
Anytime	201 (50.9%)

n (%); * A multiple-response item.

**Table 3:** Factors and predictors of nicotine pouch use (n = 489).

	Nicotine Pouch Use	Multivariable Regression				
Characteristic	No N = 94	Yes N = 395	*p*-Value	OR	95% CI	*p*-Value
**Gender**			<0.001			
Male	69 (15.3%)	381 (84.7%)		Reference	Reference	
Female	25 (64.1%)	14 (35.9%)		0.17	0.07, 0.38	<0.001
**Nationality**			0.714			
Saudi	89 (19.4%)	370 (80.6%)		Reference	Reference	
Non-Saudi	5 (16.7%)	25 (83.3%)		1.48	0.52, 5.03	0.492
**Age**			<0.001			
18 to 25	53 (21.6%)	192 (78.4%)		Reference	Reference	
26 to 35	8 (6.2%)	121 (93.8%)		2.23	0.98, 5.62	0.069
36 to 45	16 (23.5%)	52 (76.5%)		0.46	0.19, 1.15	0.095
46 to 55	12 (33.3%)	24 (66.7%)		0.26	0.09, 0.74	0.011
Above 55 years	5 (45.5%)	6 (54.5%)		0.15	0.03, 0.77	0.019
**Marital status**			0.622			
Married	35 (18.1%)	158 (81.9%)		Reference	Reference	
Unmarried	59 (19.9%)	237 (80.1%)		0.71	0.34, 1.46	0.357
**Education**			0.161			
Not educated	1 (9.1%)	10 (90.9%)		Reference	Reference	
High school student	17 (28.8%)	42 (71.2%)		0.31	0.02, 2.03	0.299
Bachelor’s Degree	68 (18.7%)	296 (81.3%)		0.30	0.02, 1.75	0.271
Master’s Degree	4 (10.3%)	35 (89.7%)		0.99	0.04, 8.89	0.990
Doctoral degree	4 (25.0%)	12 (75.0%)		0.60	0.03, 5.87	0.691
**Smoking status**			<0.001			
Never smoked	39 (44.8%)	48 (55.2%)		Reference	Reference	
Sometimes smoked	25 (15.3%)	138 (84.7%)		3.65	1.84, 7.35	<0.001
Every day smoke	30 (12.6%)	209 (87.4%)		4.57	2.40, 8.76	<0.001

n (%); Pearson’s chi-squared test; Fisher’s exact test; Abbreviations: CI = Confidence Interval, OR = Odds Ratio. A reference category in logistic regression is a baseline category for a categorical predictor variable to which all other categories are compared.

**Table 4:** Health effects of nicotine pouch use among users (n = 395).

Characteristic	Univariable Regression		Multivariable Regression		
	Health Effects Score *	*p*-Value	Beta	95% CI	*p*-Value
**Gender**		0.830			
Male	13.0 (8.0, 20.0)		Reference	Reference	
Female	14.5 (10.0, 18.0)		1.69	−3.01, 6.39	0.479
**Nationality**		0.250			
Saudi	13.0 (9.0, 21.0)		Reference	Reference	
Non-Saudi	12.0 (5.0, 19.0)		−0.13	−1.74, 0.52	0.250
**Age**		0.102			
18 to 25	13.0 (8.0, 18.0)		Reference	Reference	
26 to 35	13.0 (10.0, 20.0)		0.34	−1.87, 2.54	0.765
36 to 45	14.5 (5.0, 26.5)		0.58	−2.54, 3.70	0.714
46 to 55	18.0 (8.0, 28.0)		1.86	−2.20, 5.92	0.368
Above 55 years	22.5 (11.0, 30.0)		1.76	−6.02, 9.54	0.657
**Marital status**		0.026			
Married	14.0 (10.0, 25.0)		Reference	Reference	
Unmarried	13.0 (8.0, 18.0)		−0.65	−2.82, 1.51	0.554
**Education**		<0.001			
Not educated	22.5 (13.0, 29.0)		Reference	Reference	
High school student	8.5 (4.0, 15.0)		−9.93	−16.0, −3.82	0.002
Bachelor’s Degree	13.0 (9.0, 19.0)		−5.65	−11.3, −0.03	0.049
Master’s Degree	17.0 (10.0, 28.0)		−3.46	−9.68, 2.75	0.274
Doctoral degree	28.5 (25.5, 29.5)		6.23	−1.24, 13.7	0.102
**Smoking status**		0.002			
Never smoked	20.0 (12.0, 29.5)		Reference	Reference	
Sometime smoked	13.0 (6.0, 21.0)		−5.70	−8.59, −2.81	<0.001
Every day smoke	13.0 (9.0, 18.0)		−5.96	−8.70, −3.22	<0.001

* Median; Wilcoxon rank sum test; Kruskal–Wallis rank sum test; Abbreviation: CI = Confidence Interval; A reference: category in logistic regression is a baseline category for a categorical predictor variable to which all other categoric are compared.

**Table 5:** Psychological effects of nicotine pouch use among users (n = 395).

Characteristic	Univariable Regression		Multivariable Regression		
	Psychological Effects Score *	*p*-Value^2^	Beta	95% CI	*p*-Value
**Gender**		0.952			
Male	7.0 (5.0, 12.0)		Reference	Reference	
Female	7.0 (6.0, 13.0)		1.17	−1.90, 4.25	0.454
**Nationality**		0.259			
Saudi	7.0 (5.0, 13.0)		Reference	Reference	
Non-Saudi	7.0 (3.0, 10.0)		−2.11	−4.48, 0.25	0.080
**Age**		0.291			
18 to 25	7.0 (4.0, 11.0)		Reference	Reference	
26 to 35	8.0 (7.0, 12.0)		0.74	−0.70, 2.19	0.314
36 to 45	8.5 (4.0, 14.5)		0.63	−1.42, 2.67	0.546
46 to 55	9.0 (2.5, 14.5)		−0.42	−3.08, 2.24	0.756
Above 55 years	8.5 (5.0, 11.0)		−0.75	−5.84, 4.35	0.774
**Marital status**		0.022			
Married	8.0 (6.0, 14.0)		Reference	Reference	
Unmarried	7.0 (4.0, 11.0)		−0.79	−2.21, 0.62	0.272
**Education**		0.005			
Not educated	4.0 (1.0, 18.0)		Reference	Reference	
High school student	6.5 (1.0, 10.0)		−1.38	−5.38, 2.63	0.500
Bachelor’s Degree	7.0 (5.0, 12.0)		0.74	−2.94, 4.42	0.693
Master’s Degree	10.0 (6.0, 14.0)		1.46	−2.61, 5.52	0.482
Doctoral degree	14.0 (10.0, 16.0)		5.60	0.71, 10.5	0.025

* Median; Wilcoxon rank sum test; Kruskal–Wallis rank sum test; Abbreviation: CI = Confidence Interval; A reference category in logistic regression is a baseline category for a categorical predictor variable to which all other categories are compared.

**Table 6:** Factors associated with mouth or gum irritation.

Characteristic	Mouth or Gum Irritation		*p*-Value
	Not at All N = 104	Slight to Extreme N = 291	
**Gender**			0.536
Male	99 (26.0%)	282 (74.0%)	
Female	5 (35.7%)	9 (64.3%)	
**Nationality**			0.109
Saudi	94 (25.4%)	276 (74.6%)	
Non-Saudi	10 (40.0%)	15 (60.0%)	
**Age**			0.105
18 to 25	58 (30.2%)	134 (69.8%)	
26 to 35	23 (19.0%)	98 (81.0%)	
36 to 45	16 (30.8%)	36 (69.2%)	
46 to 55	7 (29.2%)	17 (70.8%)	
Above 55 years	0 (0.0%)	6 (100.0%)	
**Marital status**			0.045
Married	33 (20.9%)	125 (79.1%)	
Unmarried	71 (30.0%)	166 (70.0%)	
**Education**			0.044
Not educated	2 (20.0%)	8 (80.0%)	
High school student	19 (45.2%)	23 (54.8%)	
Bachelor’s Degree	74 (25.0%)	222 (75.0%)	
Master’s Degree	8 (22.9%)	27 (77.1%)	
Doctoral degree	1 (8.3%)	11 (91.7%)	

n (%); Fisher’s exact test; Pearson’s chi-squared test.

**Table 7:** Factors associated with mouth infection.

Characteristic	Mouth Infection		*p*-Value
	Not at All N = 192	Slight to Extreme N = 203	
**Gender**			0.515
Male	184 (48.3%)	197 (51.7%)	
Female	8 (57.1%)	6 (42.9%)	
**Nationality**			0.950
Saudi	180 (48.6%)	190 (51.4%)	
Non-Saudi	12 (48.0%)	13 (52.0%)	
**Age**			0.005
18 to 25	111 (57.8%)	81 (42.2%)	
26 to 35	50 (41.3%)	71 (58.7%)	
36 to 45	22 (42.3%)	30 (57.7%)	
46 to 55	8 (33.3%)	16 (66.7%)	
Above 55 years	1 (16.7%)	5 (83.3%)	
**Marital status**			0.002
Married	62 (39.2%)	96 (60.8%)	
Unmarried	130 (54.9%)	107 (45.1%)	
**Education**			<0.001
Not educated	2 (20.0%)	8 (80.0%)	
High school student	28 (66.7%)	14 (33.3%)	
Bachelor’s Degree	146 (49.3%)	150 (50.7%)	
Master’s Degree	16 (45.7%)	19 (54.3%)	
Doctoral degree	0 (0.0%)	12 (100.0%)	
**Smoking status**			0.018
Never smoked	15 (31.3%)	33 (68.8%)	
Sometime smoked	65 (47.1%)	73 (52.9%)	
Every day smoke	112 (53.6%)	97 (46.4%)	

n (%); Pearson’s Chi-squared test; Fisher’s exact test.

## Data Availability

The raw data supporting the conclusions of this article will be made available by the authors on request.
